# Perspectives of Family Members on Using Technology in Youth Mental Health Care: A Qualitative Study

**DOI:** 10.2196/mental.7296

**Published:** 2017-06-23

**Authors:** Shalini Lal, Winnie Daniel, Lysanne Rivard

**Affiliations:** ^1^ University of Montreal Hospital Research Center University of Montreal Montreal, QC Canada; ^2^ School of Rehabilitation Faculty of Medicine University of Montreal Montreal, QC Canada; ^3^ Douglas Mental Health University Institute McGill University Montreal, QC Canada; ^4^ CIUSSS du Nord-de-'Ile, Albert-Prévost Mental Health Hospital Montreal, QC Canada

**Keywords:** family, adolescent, young adult, technology, telemedicine, mental health services, psychotic disorders

## Abstract

**Background:**

Information and communication technologies (ICTs) are increasingly recognized as having an important role in the delivery of mental health services for youth. Recent studies have evaluated young people’s access and use of technology, as well as their perspectives on using technology to receive mental health information, services, and support; however, limited attention has been given to the perspectives of family members in this regard.

**Objective:**

The aim of this study was to explore the perspectives of family members on the use of ICTs to deliver mental health services to youth within the context of specialized early intervention for a first-episode psychosis (FEP).

**Methods:**

Six focus groups were conducted with family members recruited from an early intervention program for psychosis. Twelve family members participated in the study (target sample was 12-18, and recruitment efforts took place over the duration of 1 year). A 12-item semistructured focus group guide was developed to explore past experiences of technology and recommendations for the use of technology in youth mental health service delivery. A qualitative thematic analysis guided the identification and organization of common themes and patterns identified across the dataset.

**Results:**

Findings were organized by the following themes: access and use of technology, potential negative impacts of technology on youth in recovery, potential benefits of using technology to deliver mental health services to youth, and recommendations to use technology for (1) providing quality information in a manner that is accessible to individuals of diverse socioeconomic backgrounds, (2) facilitating communication with health care professionals and services, and (3) increasing access to peer support.

**Conclusions:**

To our knowledge, this is among the first (or the first) to explore the perspectives of family members of youth being treated for FEP on the use of technology for mental health care. Our results highlight the importance of considering diverse experiences and attitudes toward the role of technology in youth mental health, digital literacy skills, phases of recovery, and sociodemographic factors when engaging family members in technology-enabled youth mental health care research and practice. Innovative methods to recruit and elicit the perspectives of family members on this topic are warranted. It is also important to consider educational strategies to inform and empower family members on the role, benefits, and use of ICTs in relation to mental health care for FEP.

## Introduction

Information and communication technologies (ICTs) such as websites, social media, and mobile phones or tablet apps are increasingly recognized as having an important role in the delivery of mental health services. Some of the benefits associated with the use of ICTs include the potential to increase access to services, reduce service-related costs and social stigma, enhance service engagement, facilitate peer support, allow rapid access to information, and facilitate communication between patients and providers [1-3]. Mohr et al [4] cite videoconferencing, standard phone interventions, Web-based interventions, mobile technologies, and virtual reality as efficient enhancers of mental health outcomes when used as a complement to existing services.

Youth are often drawn to the Web during the onset of mental health issues or while receiving services [5,6]. Accordingly, in the early psychosis intervention literature, there are recent examples of studies evaluating young people’s access and use of technology, as well as their perspectives on using technology to receive mental health information, services, and support [6-8]. However, limited attention has been given to the perspectives of family members in this regard.

Family members have an important role in the management, treatment, and outcomes of youth with psychosis [9,10]. Their participation in the treatment process has been associated with a significant reduction on the risk of relapse and hospitalization [11] and with an increase in compliance to treatment and medication [10]. Thus, as ICTs are increasingly introduced into the youth mental health care system, it will be important to involve family members, for example, by better understanding their perspectives on this subject matter. Consequently, this study aimed to explore the perspectives of family members on the use of ICTs to deliver mental health care to youth within the context of early intervention services for psychosis.

## Methods

This study used a qualitative approach where family members of youth receiving services for a first-episode psychosis (FEP) were recruited to participate in focus group discussions on the topic of technology in relation to mental health service delivery.

### Recruitment

A total of 12 participants (target sample was 12-18) were recruited from a specialized early intervention program for psychosis located in an urban Canadian setting. The program provides medical and psychosocial assessments and treatments to young people between the ages of 14 and 35 years who have experienced FEP, as well as monitoring and support to their family members. The clinical team screened potential participants for eligibility from the list of family members of young people currently involved in the program. Participants’ whose family member was going through a crisis or was hospitalized at the time of the study were excluded. A member of the research team contacted interested participants, explained the project, confirmed eligibility, and inquired about availability to participate in a focus group. Written informed consent was obtained from all participants prior to participating in the study. This study was approved by the affiliated university-based ethics review board, and was part of a larger project on access and use of technology for mental health information, services, and support within the context of early intervention for psychosis.

### Procedure

A total of 6 focus groups were conducted, with the number of participants in each group ranging from 1-4. Whereas the initial aim was to have 3 to 4 participants in each group, on some occasions, participants confirmed attendance but did not attend the scheduled meeting. In this case, if only 1 or 2 participants attended, the meeting was still held, and those who were unable to attend were invited to a subsequent meeting.

Focus groups were cofacilitated by 2 members of the research team and the duration of focus groups ranged from 90-120 min. A 12-item semistructured interview guide was constructed by the first author with additional input from the second author and was used to lead the focus groups. Examples of questions asked were: Have you used technology in the past to access mental health information, services, or support; if yes, what type of information or services did you search for? Did you find the results helpful? What are your thoughts on using technology to deliver mental health information, services, and support at (name of program)? What recommendations do you have for the use of technology in the delivery of mental health services for youth?

Each focus group session was audio-recorded and transcribed verbatim. The data was managed using Atlas.ti (Version 7.5.6) and the analysis and coding approach was informed by Braun and Clarke [12]. Codes were grouped in relation to similarity, categorized, and analyzed to assign meaning. An initial coding framework was developed based on transcripts from the first 3 focus groups, which closely reflected the topics addressed in the interview guide. The framework was further developed through review of the remaining transcripts and discussion with members of the research team. The coding framework was then piloted on 4 transcripts by 2 members of the research team, revised based on team consensus, and then systematically applied to all the data. The final framework consisted of 4 primary categories with respective subthemes: experience with services, not related to technology; experience with the use of technology; concerns; and recommendations. Following Braun and Clark’s process [12], the qualitative thematic analysis was independent of theory and focused on the identification and organization of common themes and patterns observed across the dataset. In this paper, we report on the analysis of content from the last 3 categories that were directly related to technology. Some of the focus groups in the study were conducted in French; in this regard, all French quotations cited in the study were first translated into English, and then back translated into French for verification.

## Results

Details on the sociodemographic characteristics of participants are provided in Table 1.

**Table 1:** Sociodemographic breakdown of focus group participants (n=12).

Characteristics		n
**Sex**		
	Male	1
	Female	11
**Age (years)**		
	48-62	8
	Unknown^a,b^	4
**Length of involvement in the program**		
	6-12 months	1
	<6 months	3
	>12 months	5
	Unknown^a^	3
**Self-reported ethno-cultural background**		
	White	10
	Black	1
	Asian	1
**Education**		
	High school completed	1
	CEGEP^c^or college completed	3
	Undergraduate completed	1
	Master degree	4
	Unknown^a^	3
**Employment status**		
	Not employed	1
	Employed	2
	Part-time	2
	Full-time	4
	Unknown^a^	3
**Living situation**		
	With the family member involved in the program	9
	Unknown^a^	3
**Relationship with patient**		
	Mother	9
	Step-Mother	1
	Wife	1
	Father	1
**Annual income (CAD$)**		
	30,000 - 49,999/year	4
	50,000/year and more	4
	Did not indicate^b^	1
	Unknown^a^	3

^a^3 participants did not complete sociodemographic information.

^b^Some participants did not reply to all the questions.

^c^CEGEP: Collège d’enseignement général et professionnel.

The results are organized into 4 topical areas: (1) access and use of technology, (2) concerns about using technology, (3) benefits, and (4) recommendations.

### Access and Use of Technology

At the start of each focus group, participants were invited to describe their experiences of using technology, such as the Web, mobile technologies, and social media. Some of the participants reported having limited experience with using ICTs, citing personal and external reasons such as lack of time, poor familiarity with devices, lack of skills, and lack of need. Most participants did report using devices such as cellphones, tablets, computers, and televisions as platforms for entertainment (eg, movies and music), for managing daily activities and appointments (eg, calendars), to communicate with peers (eg, through chat rooms, forums on mental health, and blogs with testimonies), and to stay in communication with family members and health care professionals (eg, through emails and texting). In this regard, they highlighted the important role that technology plays in their daily lives:

My tablet is associated with leisure, games, or things like that. And my computer is associated with my work. Which means that...But, except that I still believe in technology.

P10

Go look for news, go look for things that are new, whether it be via Twitter, via my emails...I would say that 80% of my interactions take place on that, whereas 15 or 20% will take place on the phone. So, technology, it’s very important.

P11

Some participants also described searching the Web for mental health information. This was even the case with participants who initially reported limited use of technology, for example, participant 2 had initially stated:

I don’t find a need in it. For me, technological tools are to be used. And, if I don’t need it, I will not go play with it just for the sake of it, to see what I can do with it.

P2

Later in the discussion, P2 explained:

I looked at many websites. I watched video-conferences on websites that address mental illness. What were the different diagnoses? Also, I looked for online questionnaires.

P2

Some mentioned that they searched for information on the Web particularly during the early course of their family member’s illness and treatment:

I had no idea what psychosis was. It took me a long time before—and that’s what I was reading about...I was just trying to collect information to see what he has.

P7

Participants also described the need to search for more information following an initial visit to the emergency room with their family member during an episode of psychosis:

Don’t have a choice. We go look for other resources because the ones that we get at the emergency do not treat the person...look here is a small piece of paper, a prescription, and bye. So then you don’t have a choice but to go look elsewhere because you tell yourself, listen, I can’t leave her like that.

P12

Thus, participants searched for information on the Web pertaining to (1) psychosis, (2) resources for symptom management, (3) emergency numbers, (4) contact information to reach health care professionals and programs, and (5) recent advancements in mental health care (eg, on hospital websites). When they managed to find this type of information, their experiences were reported as being positive.

### Concerns

Participants described several issues, challenges, and concerns encountered in their past use of technology. For example, one participant described how despite their best efforts, they were never able to find the information they were looking for:

Then, when our son had his episode, it’s the first thing that I did, I went on the Internet, I went to see the website of the (local mental health institute). I really looked and looked. I didn’t find what I wanted, but I looked. When I have a problem of some sort, it’s my reflex. It’s to go on the Internet.

P10

Participants also highlighted specific concerns related to the negative impact of information on the Web on young people’s mental health, coping, recovery, and treatment process. For example, one participant described how information on the Web affected adherence to medication:

He goes on the Internet too, though. He’s obsessed with looking up his symptoms...Then, if he finds an article that says that the pills aren’t good, I tell you he’s quick to throw it back at you. “Look, mom, the pills, that’s what they do, that’s what they can do to me, that’s what they can do to me.”...Then, he tells me, look, this is serious. There’s research on this, this can cause me damage.

P8

Another participant described how information on the Web influenced the use of substance abuse:

He’s often on the Internet. Yes, he always wants me to read something, listen to something, everything that talks about pot, and the benefits of pot. Yes, he is very convinced of that. He goes looking for that on the Internet. Which means that for me, personally, access to information is great, but there is a limit.

P9

Others explained that Web-based interactions and communications can exacerbate social isolation. In this regard, they emphasized the value of reconstructing a social life in person as opposed to using social media, chat rooms, or Web-based dating services:

It takes face-to-face human contact. It takes someone who will motivate your youth. Technology, it’s cold.

P9

Another participant argued that the use of Web-based technologies might worsen psychosis:

Especially if you already have mental health problems, if you have suffered a psychosis in which reality was not really reality, your reality. And now you’ll get into a situation where everyone is not quite real, is virtual. (...) It’s even worse. For me, it can disturb more the state of mind...I think it might even affect normal people, eventually.

P2

This same participant also raised the issue that young people with psychosis may not have the abilities to communicate effectively online because of apathy, anxiety, social withdrawal, lack of initiative, and decreased cognitive capacities.

Participants also highlighted how the use of certain ICTs might increase the potential for deception, misunderstanding, and abuse:

You know, you cannot read the unsaid in a chatroom, you know (...) You cannot see the nonverbal...It puts you on the wrong track, it can really lure you (...) you might think that this person here is the best person, the best friend in the world, but they might in fact be the worst.

P1

Participants also raised concerns about the ability to manage the use of technology:

The evening of his crisis...He hadn’t charged (his cellphone). He had forgotten to charge it, which means that it was in his pocket but it wasn’t charged...He was lost in (urban city), but anyways, we ended up finding him...

P8

Other issues and concerns participants raised related to the (1) relevance, quality, and source of mental health information, (2) addictive and time consuming nature of technology, (3) accessibility and reliability of technology (eg, unaffordability, Internet not working, thefts), (4) privacy and confidentiality, and (5) website characteristics (eg, interactive and user-friendly features, accessible nonmedical language).

### Benefits

All participants expressed receptivity to the idea of using technology in relation to access to mental health information, communication with service providers and youth, and access to education and peer support. In terms of access to mental health information, one participant highlighted:

If you accumulate all the information and put it in the website...I think it will have a lot of positive aspects in the long run that could save a lot of money, that could save clients, that could save caregivers, that could save the family dynamics.

P7

Participants also spoke about how Web-based information could help families anticipate and respond to a young person going through early stages of mental illness:

If there was a website where I could go in the beginning—right in the beginning where I called, right before he was really in psychosis. But if there was a website where I could say, you know, “XXXX, come sit with me. Look at this. We’ve got a plan over here. You know, we need to go to the hospital. This is what’s going to happen in the hospital. Now if you agree or disagree, if you get ill or whatever, these are the legal steps in (city) that happens here,”...more like an education, you know?...all the things that are going to be happening to them, to their loved ones, whoever it is, it’s like they need to have a sort of a picture of what they’re looking at.

P7

They emphasized that access to information on the Web was relevant because they did not want to overburden their child with questions:

I’m afraid to ask too many questions, if it’s going ok? And, the medication, does it make you gain weight? Does it make you sleep? I don’t want to bother him too much with that, you know...but if there are places where he can go and ask his own questions, and satisfy his curiosity, and that they are easily referenced, that could be interesting.

P11

They also explained the benefits of technology for educating other family members and the public to help with stigma, mental health literacy, and reduce misconceptions:

It’s friends and family who understand absolutely nothing. They ask all sorts of questions. They want to know is he schizophrenic or is he not schizophrenic? It comes back to that a lot.

P11

It will reduce stigma...because anyone will be able to access it, it will go very fast...They’ll go on our website and they say “Oh, I did not know that! That’s what Johnny does at school. I’ll contact his parents to watch it”...Something that is understandable, that is clear and that can be seen by anyone. I think it has tremendous benefits, you know.

P2

In addition, they described that it can support a young person in managing symptoms and other related issues, such as substance use:

I think, for the kids, the children should have a website that should show them the early signs of taking these drugs—what can happen to them, to their brain cells and to the side effects of these drugs.

P4

Finally, they believed that the use of Web-based technology could help health care providers remain up-to-date with new developments: *Like your website could help the doctors, the psychologists, you know? Because a lot of doctors, they’ve become doctors from a while back.* [P7]

In terms of facilitating communication with service providers and youth, they highlighted that this was particularly helpful during times of social withdrawal and isolation, for example, through texting:

She had isolated herself from all of her friends and all of that. She wasn’t seeing anyone anymore. She wasn’t going to school or to work. Which means she had completely isolated herself. Me, the fact that I was in the last few people, I was ok for texting, and she would respond, but I felt like it was holding by a thread...the thing I like as well about texting, it’s that they are free to respond when they want to...it attracts attention too, and they feel like they are supported too.

P12

In terms of the use of Web-based technologies to promote participation in family psychoeducation groups and peer support, participants highlighted how this would promote disclosure and be particularly helpful during winter weather:

Imagine that...because if you’re not in front of the other person, you are able to reveal more things. So, I would think the only way you could do that would be on a Skype type thing. Like to have a virtual meeting, to have a virtual meeting, of course, it’s more convenient for people, they don’t have to get out at -20 in the middle of January.

P2

### Recommendations

Participants provided several recommendations in terms of future use of technology in youth mental health care, which could be categorized into 3 subthemes: (1) provide quality information in a manner that is accessible to individuals of diverse socioeconomic backgrounds, (2) facilitate communication with health care professionals and services, and (3) increase access to peer support. Each of these themes are elaborated in the following sections with examples of illustrative quotes provided in Table 2.

#### Providing Quality Information in an Accessible Manner

Participants explained that providing information about the services offered in an accessible and inviting manner would boost comfort in accessing mental health care services. They emphasized that Web-based information needs to be accessible to individuals across all levels of education, experience with mental illness, or experience with the mental health care system. Toward this end, they suggested providing information through a variety of tools and formats, such as movies, educational videos, websites, forums, and mobile phone apps. Moreover, they insisted that regardless of the means or tools, the primary goal of health care is to provide a common language for youth, family members, and service providers. Participants also highlighted the opportunity for using technology to provide information on topics that are important for youth to know but are not necessarily prioritized during clinical meetings (eg, sex education).

#### Facilitating Communication With Health Care Professionals and Services

Participants made several suggestions on the use of technology to facilitate communication between youth, family, and health care providers, such as using shared calendars, having appointments online, using apps with a sharable journal of symptom tracking and management strategies, online forums, and chat rooms for clients and family members. They perceived the use of ICTs as complementary to in-person services provided by trained health care professionals. They cautioned, however, that if too much information and interaction was provided on the Web, people might not see the need to attend meetings with health care professionals.

#### Increasing Access to Education and Peer Support

Participants suggested that technology could be used to increase access to education and peer support. For example, psychoeducation could be accessed at home through webinars or prerecorded conferences. They highlighted that the latter could increase participation for people who might face challenges in attending and engaging in family support and education sessions that are typically only offered in-person. Participants also offered suggestions in relation to using technology to facilitate opportunities to receive peer support (either for family members or youth). To this end, some suggested volunteer peer-mentorship for clients and family members to help support newly diagnosed clients and their families. Some participants also suggested including contact information of volunteer clients and family members who would be willing to be mentors and provide support to newly diagnosed clients and their family members. They argued that a positive experience of service delivery supported by peer interaction could be beneficial to the service provider’s image and reputation, as people will likely recommend the services with which they were satisfied. Key messaging should be to reduce misconceptions about mental health and provide support to families.

**Table 2:** Participants’ recommendations.

Subthemes	Participant quote
Providing quality information in an accessible manner	“If we could write somewhere ‘We are here to help you, we will find solutions,’ then it would be so comforting. Sometimes just a little phrase to say, you know, we’re here! As if to provide a bit of soothing...just be a little bit less medical and cold, you know, ‘That’s it, then that’s the disease.’ And, perhaps, use more words, a little more vulgarized [ie, accessible language].” [P1]
	“If we want to go get a larger clientele, they are not all university graduates. Which means that, it has to be simple sentences...Not too busy either, on the webpages.” [P12]
	“Capsules of 15, 20, or 30 minutes, maybe not more than 20 minutes, 3 or 4 capsules on that and, this will help enormously because there is a big problem with that...I think you have all of the material to do this. And with technology, you can connect all this together.” [P11]
Facilitating communication with health care professionals and services	“eHealth must be an additional service and it should encourage, either to the family or for customers, us, to get in touch with you.” [P2]
	“And we mentioned lots of things: a website, chatrooms, SMS...” [P11]
Increasing access to education and peer support	“Peer support. Those who are ready to say: ‘Ok, me, I could be listed on a call bank.’ ...Then, say, ‘I’ve been through what you have been through, you know. Then, on the phone, we can talk, we can go grab coffee together, if it’s not too far for you.’ You know, make it friendly and based on the human person.” [P1]
	“To let them know that they are not alone—to let them know that mental illness is not something that is to be held against a person, that is not necessarily a...hum...a negative thing. It is just something within the person itself that can also come from a physical, biological, chemical imbalance.” [P6]

## Discussion

### Principal Findings

To our knowledge, this is among the first (or the first) study exploring the perspectives of family members receiving services from a prevention and early intervention program for FEP on the use of technology within the context of mental health care. Participants faced several barriers in accessing mental health services, information, and support on the Web, such as lack of time, digital literacy skills, perceived need, and lack of affordability of ICT devices. However, both regular and occasional users identified a range of actual and potential benefits, as well as recommendations for using ICTs in youth mental health care. Research on the perspectives of individuals with diverse experiences and attitudes toward technology is warranted for the future development and implementation of ICTs in youth mental health care.

Participants’ use of ICTs to support their family member is similar to those reported in the broader literature. For example, a review conducted by Park et al on health-related Web use by informal caregivers of children and adolescents, identified searching the Web for “disease specific information about disorders and treatments” and seeking “social support for emotional needs via a virtual community” as the most common use of the Web [13]. Moreover, in line with the barriers raised by participants about the importance of digital literacy and sociodemographic factors to access and use ICTs, Park et al also identified a digital divide linked to social status (racial and ethnic minorities) and education level in relation to Web access [13]. Furthermore, previous work by Klee et al [14] demonstrated that sociodemographic barriers can impede access to technology for individuals coping with mental illness and our study illustrates that this may be relevant for family members as well. Since mental health care settings and services can already be difficult to access and navigate, it is important that ICTs in this context help bridge the gap between family members and providers instead of widening it because of a lack of digital literacy or economic means.

Benefits identified by family members were consistent with perspectives obtained from youth in previous research. For example, Lal et al [6] found that youth valued the use of ICTs to access information and peer support to help cope and manage with mental health concerns. Concurrently, some of the concerns raised by family members were different than those identified by youth. For instance, family members in this study expressed concerns about the capacity of young people with psychosis to use and manage technologies, how ICTs could be addictive and time consuming, negatively impact treatment adherence, social recovery, and contribute to substance abuse; whereas youth in previous research placed more emphasis on concerns related to the quality, reliability, and trustworthiness of information available on the Web [6]. However, both family members and youth from previous research highlight the importance of safeguard measures, specifically the professional role in the use of ICT tools and platforms related to mental health care.

### Implications for Practice

Family members highlighted several benefits and made specific suggestions around the use of ICTs to communicate with service providers. These suggestions and tools can inform and guide clinicians and researchers who wish to engage with family members via ICTs. Providing up-to-date quality information in an accessible language was of importance to participants as they emphasized that the objective of mental health care was to ensure communication between youth, family members, and service providers. Within the broader health literature, Park et al recommend that Web-based information targeting informal caregivers be “evidence based and written at a sixth-grade level” [13]. Participants in our study suggested using a variety of formats including movies, educational videos, website forums, and mobile phone apps to increase engagement with mental health care services. Those participants that had limited use of technology also highlighted the importance of providing this information in both digital and print formats. In addition, participants listed tools to better reach and stay in contact with the clinical team, such as shared calendars, online appointments, sharable symptom tracking and management journals, as well as chat rooms and forums.

A second implication for practice included the concerns raised by participants that specifically relate to youth with psychosis. These perspectives of participants warrant further attention as they can limit the uptake of technology-enabled tools provided by specialized early intervention programs and discourage youth and family members from going on the Web to access mental health information, services, and support. Thus, it is important to consider educational strategies to inform and empower family members on the purpose, benefits, and use of ICTs in relation to mental health care for FEP. This education could be added as a component to psychoeducation workshops that are typically provided by health care providers in specialized early intervention programs. To address participants’ concerns over potentially abusive or dangerous social interactions online for an at-risk population, family members may benefit from a discussion on evolving psychosocial behaviors that are defined differently by youth. For instance, a family member may interpret a young person spending “all day on the phone” as social withdrawal, whereas the young person may be using the phone to connect and exchange with formal or informal peer supporters. Likewise, a Web-based social interaction with a peer supporter may be more beneficial than a face-to-face interaction with a service provider that is asking routine or predetermined questions and who may be pressed for time [15]. Furthermore, mental health care professionals can draw from research on cyberbullying and online harassment to develop tools and safeguard measures to help youth recovering from FEP identify and appropriately respond to potentially problematic situations [16,17]. Providing strategies on cyberbullying to family members and youth also links to family members’ recommendation to use technology to inform clients on topics not generally discussed in mental health care settings but that have a direct impact on recovery and well-being, for example sex education.

### Future Research Directions

Future research could focus on family members’ perspectives on the use of ICTs to support youth mental health care using a larger sample size. The latter could highlight the differences and similarities with the general population as well as compare responses between participants who regularly use ICTs and those who do not. A better understanding of the differences between heavy and nonusers may provide insight on accessibility and uptake of ICTs in mental health care service delivery. Moreover, building on previous studies conducted with youth [6] and the current study conducted with family members, it is important to also explore mental health care professionals’ perspectives (benefits, concerns, and recommendations) on the use of ICTs to deliver mental health services to youth recovering from FEP and other mental health problems. A reflection and discussion on how to address and integrate the needs, concerns, and objectives identified by patients, family members, and health care professionals is warranted to inform the development and implementation of ICT tools in youth mental health service delivery. The challenge in developing and implementing new ICT tools will be to effectively assess and respond to needs that are specific to each stakeholder as well as facilitate information sharing and communication among the 3 groups.

As family members strongly emphasized the need for Web-based platforms that can facilitate peer interaction in a safe context, further investigation could explore elements of Web-based platforms that increase or decrease feelings of safety and develop efficient safeguard measures. Similarly, Green et al [18] emphasize the importance of using tailored age-appropriate approaches that reflect youth cultures and lifestyles to engage youth in mental illness treatment, to increase their autonomy to manage their condition, and to improve social integration. In developing and implementing such approaches, it is also important to consider the various phases of illness and recovery pertaining to psychosis and how technology may be experienced differently during these different phases. This is an area that needs further research and innovation in terms of technologies as well as guidelines for using them.

### Study Limitations

The small sample size of 12 participants limits the generalizability of the results. Although our original target sample was small (12-18), recruitment was challenging. Most family members contacted declined to participate in the study and some cancelled their engagement prior to the focus group or did not show up after confirming attendance. A possible explanation is the topic itself. The use of technology-enabled interventions and services targeting family members within the field of early intervention for psychosis (and other areas of mental health) is still in a stage of infancy and thus, potential participants may have not felt able to contribute meaningfully to the study. We had previously conducted a focus group research project on the topic of relapse recruiting from the same setting and experienced minimal challenges in terms of recruitment. Thus, given limited exposure to the topic of technology within the context of interacting with youth mental health services, potential participants may have perceived the topic as being irrelevant to their experiences of health care services or of low personal interest. The negative attitudes or concerns regarding the impact of technology on young people’s mental health, may have also influenced family members’ interest to participate in the study. There were also challenges in coordinating participants’ schedules, even though groups were offered both in the afternoon and evening. Future research might consider recruiting participants from a broader range of sociodemographic characteristics as well as using surveys, individual interviews, or other innovative methods, rather than focus groups to better accommodate family members’ time constraints and multiple obligations.

### Conclusions

This study described the perspectives and experiences of family members on the use of ICTs to deliver mental health care services to youth who have experienced FEP. The findings support the importance for future attention on the development and testing of a variety of technology-enabled tools and services to better meet the needs and preferences of family members in their efforts to understand, manage, and cope with psychosis and related concerns. Results also highlight the importance of mental health care providers to educate family members on technological tools, platforms, and resources currently available to support psychosis management and recovery. More information is needed on family members’ perspectives to better inform the use of technology in the delivery of mental health care.

## Abbreviations

FEP: first-episode psychosis
ICTs: information and communication technologies
